# The Influence of Different Butter Type, Their Fatty Acid Composition and Melting Enthalpy on the Viability Rate of *Lacticaseibacillus* *rhamnosus* GG Directly After the Spray-Drying Process and During Storage of Powders

**DOI:** 10.3390/foods13233803

**Published:** 2024-11-26

**Authors:** Alicja Fedorowicz, Artur Bartkowiak

**Affiliations:** Center of Bioimmobilisation and Innovative Packaging Materials, Faculty of Food Sciences and Fisheries, West Pomeranian University of Technology in Szczecin, Klemensa Janickiego 35, 71-270 Szczecin, Poland; artur-bartkowiak@zut.edu.pl

**Keywords:** *Lacticaseibacillus rhamnosus* GG, spray-drying, butter, microencapsulation, protection, viability, melting enthalpy, fatty acids

## Abstract

The present work reports on the microencapsulation of *Lacticaseibacillus rhamnosus* GG (LGG) by the spray-drying process using a solution of starch, whey protein concentrate (WPC), soy lecithin and ascorbic acid as a carrier, with addition of different types of butters. The aim of this study was to examine the protective mechanism of six different butter samples on the viability rate of LGG bacteria directly after the spray-drying process and during storage for 4 weeks at 4 °C and 20 °C (±1 °C) based on hypothetical factors—the fatty acid’s chemical character and content, and its melting enthalpy. The viability of bacteria, moisture content, water activity, color properties, morphology, particle size of powder, melting enthalpy of butters and their fatty acids composition were evaluated. It is assumed that the highest viability may be indirectly influenced by the relationship between the highest content of proteins and sugars and the lowest content of fats and fatty acids, which is characteristic for butter with a reduced fat content. This butter contained also the least monounsaturated and polyunsaturated fatty acids. The highest number of viable LGG (for systems with reduced-fat butter, as well as salted and lactose-free butter) may be caused by (among other factors) by the lower content of palmitic acid (C16: 0). For these butters, it was also observed that cell viability increased with the increase in melting enthalpy. The results confirmed the protective role of selected butters, which indicates the possibility of using them in industrial processes to increase the durability of additives and products using probiotic powders obtained by spray-drying.

## 1. Introduction

The spray-drying (SD) process is one of the most popular, suitable, cost-effective and fast techniques to produce powder from liquid raw materials. This process is one of the most promising due to its ability to produce dry probiotic microcapsules and possibility to protect and improve the viability of probiotic bacteria [1]. SD is one of the encapsulation types that can be used for the delivery of probiotic cells. Encapsulation is a process in which bacteria are surrounded/encapsulated by a protective continuous film of a wall material [2].

The following stages of spray-drying are distinguished: (i) atomization of a solution/emulsion into fine droplets, (ii) contact of the resulting droplets with a hot gas medium such as air with simultaneous water evaporation, and (iii) separation and collection of the spray-dried powder [3].

### 1.1. Spray-Drying of Probiotic Bacteria

One of the major disadvantages of the spray-drying process of bacteria is the loss of activity. It is considered that two mechanisms are responsible for the inactivation of microorganisms during spray-drying: fast dehydration and high temperature [4]. It is uncertain which is more damaging, as they usually occur simultaneously. Heat inactivation theory posits that heat inactivates key elements, but also destroys many other components of living cells. The loss of less important elements does not directly cause bacteria death. However, when these “weakened” components are additionally exposed to unfavorable factors, cell deactivation may occur [5,6]. The second mechanism of bacterial cell inactivation is related to the removal of water from their interior, which leads to the formation of numerous physiological limitations including change in their physicochemical character [6]. The cytoplasmic membrane is considered the most susceptible to dehydration because its damage causes the loss of basic life functions. The structure of the lipid membrane bilayer is thermodynamically unstable and is, therefore, thought to be damaged by dehydration [7,8].

Moreover, the viability rate of the spray-dried probiotic bacteria is influenced by many factors, such as the type of bacterial strain, its growth phase, inlet and outlet air temperature, time of contact with hot air, type of dissolved substances (and thus coating material) and their total content, pH value of drying medium, spray rate and dryer design, as well as mechanical, oxidative and osmotic stress [9,10].

One of the most important factors influencing the lability of bacteria is the outlet temperature, and not the temperature of the inlet air, which typically is much higher than boiling temperature of water [11]. The outlet air temperature is an experimental variable that cannot be directly set on the device. It is a function of other factors, such as gas inlet temperature, gas flow rate, concentration of solid substances in the drying formulation and enthalpy of evaporation [12]. Due to the intense heat and mass transfer and loss of moisture, the dried particles can reach the maximum temperature of the drying gas at the dryer outlet. In the first stage of spray-drying, the drying rate is very high; this is when most of the moisture evaporates from the dried particles. On the other hand, during the second stage when the outlet air temperature affects the particles to be dried, the drying rate decreases quickly, so that more time is needed to achieve relatively low moisture content in the final product than in the first stage [13]. Reducing the inlet temperature results in higher viability after the spray-drying process, while in return leading to higher humidity and water activity of the final powder, which adversely affects bacterial viability during storage [14].

It is believed that the main factors causing bacterial death during and after the spray-drying process are thermal, osmotic, oxidative and desiccant stress. Variable degrees of tolerance to such stresses are shown by different species and even strains of bacteria [15]. It should be remembered that the thermal resistance of bacteria to high temperatures is also an individual characteristic of each bacterial strain [16]. It has been proven by scientists in the year 2000, who observed the higher viability rate of spray-dried *L. paracasei* NFBC 338 bacteria (approx. 50%) compared to *L. salivarius* UCC 118 cells (viability rate approx. 1%), which were dried under identical conditions [17]. Other researchers showed that the toleration of the drying process by *Bifidobacterium breve* is worse (viability rate only 1.4%), compared to *Bifidobacterium longum* (viability 25.1%), which were both dried under the same conditions [18].

The type of coating-forming substances also has an important impact on the viability rate of the probiotic cells. Such a material should meet the following criteria: high protection of the active ingredient, good solubility in water and the ability of film formation with of water evaporation, good emulsifying properties and low cost [19]. Film-forming substances used to protect living probiotic cells must not be toxic to them and not have bacteriostatic or bactericidal effects [20]. Complete loss of viability of *Lactobacillus zeae* LB1 was noted during spray-drying using water alone without the wall material [21]. Therefore, various coating agents and protectants such as trehalose, reconstituted skim milk (RSM), gelatin, gum arabic, sweet whey, modified starches, maltodextrin or whey protein isolate are often used during the spray-drying processes [8,22,23,24,25,26].

The protective effect of the polysaccharides is related to their ability to form a highly viscous glassy matrix during dehydration, thanks to which it remains in a metastable state [27]. Dairy-based materials are often used as probiotic protective substances due to their compatibility with probiotic bacteria and the ability to promote their effectiveness [28]. Reconstituted skim milk (RSM) is a commonly used substance [8]. Proteins in RSM may prevent damage by stabilizing cell membrane components. They can also form a protective coating on the bacterial wall when they react with milk calcium [29]. Gum arabic protects against dehydration of cell components and stabilizes bacteria during drying [30]. In the year 2015, scientists investigated and confirmed that low-melting-point fats (LMF) protect *Lactobacillus* bacteria from damage when added to sodium caseinate during spray-drying. This protection may be related to the fact that LMF absorbs part of the thermal energy, which positively affects the probiotic cells during the spray-drying process [21].

Not only is the type of substances used important, but also their content. According to reports in the literature, dry matter content in the solution in which the bacteria are suspended is usually between 20 and 30% (*w*/*v*) [31,32,33,34]. Thanks to the high total solids content, the efficiency of the drying process is higher, energy cost is lower, and as result of all the final effect of encapsulation is better [35]. However, the higher the solids content, the bigger the size of dried particles, which increases the contact time between the hot drying gas and the dried material and thus thermally inactivates the bioactive material [6].

The growth phase of probiotics also affects the resistance of probiotics to process conditions. They are generally harvested until the late exponential or early stationary phase. However, the stationary phase is more often mentioned as optimal because cells in this phase show higher viability during drying processes [36]. In 2004, researchers studied the effect of the growth phase of LGG and the addition of prebiotics on the viability rate of these bacteria during spray-drying. Using a 20% (*w*/*v*) powdered milk solution, the highest bacterial viability rate was obtained in the stationary growth phase, it was lower in the early phase of logarithmic growth, and the lowest in the lag phase. However, after using a mixture of powdered milk (10% *w*/*v*) and polydextrose (10% *w*/*v*), the spray-drying process was most effective in terms of the bacteria viability in the early log phase, to a lower extent in the stationary phase, and the least in the lag phase [31].

Bacteria viability is also influenced by the feed rate of the dispersed material, which is defined as the inlet volume flow (ml/min) of the liquid stream. The feed rate in respect to type of atomization method and gas flow rate affects on the powder size and moisture content. The higher the flow rate of the raw material, the larger the atomized particle size of the final product obtained, as well as the moisture content of the powder. Low atomization speed results in low moisture content of powder, while high suction speed results a higher degree of separation inside the cylindrical blanket [12]. Drying time is the time from the entry of the sprayed liquid into the drying chamber to its exit from the dryer as a powder [37]. The type of device (e.g., its size) also affects the viability of sensitive microorganisms during the spray-drying process. Here, the main factor is probably the residence time of the bacteria in a hot drying chamber: the longer the residence time, the lower the viability rate [35]. However, this does not seem to be the general rule as other researchers showed no significant differences in bacterial viability after spray-drying at the laboratory and at the pilot scale [38].

### 1.2. Powder Storage

In addition to the above-discussed process parameters, the degree of viability of spray-dried cells during storage is influenced by various factors. Namely, they are storage temperature, residual moisture, relative humidity, oxygen content, exposure to light, the type of protective substance applied and the size of powder particles [39]. The moisture content of the obtained powders is a significant parameter affecting the viability of microorganisms during storage. Too low powder moisture, in the range of 0.2–1.2%, can cause cracks in the dried rigid particles, contributing to the oxidative degradation of proteins and cellular compounds [40]. In 2004, researchers noted that at a moisture content below 7%, the diffusion of water through the food matrix is reduced, which lowers the effect of moisture on the physical and chemical properties of the solid matrix [41].

Water activity is an important indicator due to its impact on the durability of the formed powder. High water activity indicates that more free water could be available for biochemical reactions and consequently, this results in shorter shelf-life [42]. When the a_w_ value is greater than 0.25, it can reduce the viability of bacteria by increasing bacterial metabolism [43]. The encapsulated material has a long shelf-life when it is stored below the glass transition temperature of the capsule matrix. This is because in such conditions the risk of bacterial growth and biochemical reactions is very low [44].

During storage, the oxidation of membrane lipids reduces the viability of microorganisms. As a result of lipid peroxidation, there is a reduction in the ratio of unsaturated to saturated fatty acids in the lipids of the cell membrane of LAB. It has been shown that during storage the lipid peroxidation products cause damage to the cell wall, cell membrane and DNA of bacterial cells. This damage can be reduced by adding antioxidants [44]. For example, vitamin C can function as an active antioxidant in bacterial cells to quench radicals [45]. In 2012, scientists showed that the addition of vitamin C to skim milk during the spray-drying of *Lactococcus lactis* subsp. *cremoris* ASCC930119 resulted in >10% higher viability [46]. The durability of the powder is also influenced by its storage method and packaging. Similar to typical spoilable food products, the most common factors that should be avoid are oxygen, moisture, light, microbial contamination and elevated temperatures. This is why the options for packaging are different types of barriers for these factors (e.g., high barrier plastic bags and blister packs) [24].

Many publications point out that the viability of probiotic cells is too low. Moreover, very often the survival and colonization of these bacteria in the human digestive tract are questionable. Therefore, there is an increasing interest in providing probiotic cells with a physical barrier that would protect them from the effects of unfavorable environmental conditions [20].

As presented above, many factors affect the viability rate of probiotic bacteria during the drying and storage process. It is known what factors reduce the viability, but to our knowledge the mechanism of the protective effect of coating substances is not known; there are no reports on evaluation of the protective effect of various butters on the viability process of probiotic bacteria during and after the spray-drying process. Commercial fatty-based food products were used in the work to verify which of them improve the viability of *Lacticaseibacillus rhamnosus* GG during drying and final storage. The aim of this study was to examine the effect of six different butter samples on the viability rate of LGG bacteria directly after the spray-drying process and during storage for 4 weeks at 4 °C and 20 °C (±1 °C). Furthermore, we have tried to verify the protective mechanism of fatty substances in the various butters based on hypothetical factors: first, the fatty acid’s chemical character and content, and second, their melting enthalpy.

## 2. Materials and Methods

### 2.1. Materials and Reagents

*Lacticaseibacillus rhamnosus* GG used as a probiotic was obtained from dietary supplement Acidolac Baby (Polpharma, Starogard Gdanski, Poland). Modified starch CAPSUL^®^ HS was purchased from Ingredion (Hamburg, Germany). It is a dedicated starch-based encapsulation system that meets, among others, food microstandards; it is designed to maximize solids before spray-drying with a solubility above 99%. Whey protein concentrate (WPC 80) was received from the Institute of Dairy Industry Innovation (Mragowo, Poland). Soy lecithin Emulfluid E was received from Cargill, and ascorbic acid was received from AQUANOVA AG (Darmstadt, Germany).

The following cow milk butters were purchased from the local market:

1. Extra—83% fat content (Mlekpol, Grajewo, Poland);
2. “Whetstone” extra—82% fat content (Pilos, Lukow, Poland);
3. Reduced—fat content 60% (Pilos Warlub, Poland);
4. Clarified (Mlekovita, Wysokie Mazowieckie, Poland);
5. Lactose-free (Pilos, Lowicz, Poland);
6. Salted (Kerrygold, Ireland).

MRS and MRS-agar were purchased from BTL (Lodz, Poland). NaCl, NaOH and glycerol were purchased from Chempur (Piekary Slaskie, Poland).

### 2.2. Bacterial Culture

For long-term storage, LGG was frozen in a 20% (*w*/*v*) aqueous glycerol/MRS (4:1) solution at −32 °C.

For spray-drying, the appropriate bacterial cell cultures were obtained as follows: a frozen culture of LGG was incubated on MRS broth at 37 °C for 22–24 h, then 1% (*v*/*v*) inoculum was transferred to fresh MRS broth and incubated at 37 °C for 22–24 h [21]. Bacterial cells were harvested by centrifugation at 2000 rpm for 10 min at 21 °C (MPW-352R, MED Instruments, Warsaw, Poland).

The supernatant was removed and the pellet was washed with 0.85% (*w*/*v*) NaCl [21].

### 2.3. Emulsion Preparation

**Figure 1:**
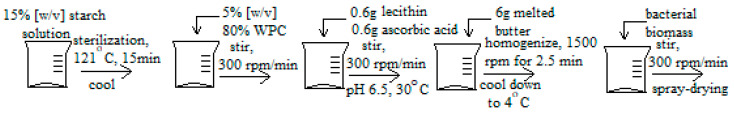
Diagram of the emulsion preparation.

A 15% (*w*/*v*) Capsul^®^ HS starch solution was sterilized in the autoclave at 121 °C for 15 min (Prestige Medical, UK), left to cool, and then 80% whey protein concentrate powder (WPC) was added in the appropriate amount (*w*/*w*) to obtain the final 20% (*w*/*v*) solution. Afterwards, lecithin and ascorbic acid (in a ratio 1:10 to final butter content) were added and pH was adjusted to 6.5 with 1M NaOH (Table 1). All substances were stirred at 300 rpm/min for around 10 min using a magnetic stirrer with heating (C-MAG HS 10 digital, IKA, Warsaw, Poland) and the obtained solution was heated to 30 °C (C-MAG HS 10 digital, IKA, Poland) to melt and disperse various butters. Then, the butters were melted at temperature in the range of 28–33 °C using a magnetic stirrer with heating and added in a ratio of 30% to the dry mass of starting dispersion and homogenized at 1500 rpm for 2.5 min using the UniDrive X1000 Drive Homogenizer (CAT, Dortmund, Germany) (Figure 1). All processes were conducted to minimize the risk of bacterial contamination. The obtained emulsions were placed in a refrigerator to cool down to 4 °C. Then, bacterial biomass was dispersed in emulsions while stirring at 300 rpm using a magnetic stirrer in the appropriate amount to obtain the final concentration of 10^9^ CFU/mL. Subsequently, the emulsions were spray-dried (Figure 1). The emulsion of starch, whey protein concentrate, lecithin and ascorbic acid without butters was used as a control sample.

Figure 1 displays a diagram of the emulsion preparation to illustrate the process.

### 2.4. The Spray-Drying Process

The spray-drying process was carried out by using a laboratory-scale spray-dryer (B-290, Büchi, Flawil, Switzerland). The spray-drying parameters were held constant during all experiments: inlet temperature of 180 °C, feed rate of 30% (around 9 mL/min), the flow of spraying air was set to 7.9 dm^3^/min and the aspirator at 80% air flow (32 m^3^/h). The outlet temperature was in the range of 47–57 °C. The spray-dried powder was collected using a single cyclone separator.

### 2.5. The Viability Rate of LGG

To determine the number of bacteria in the emulsions, ten-fold serial dilutions were performed and then plated in duplicate on MRS agar [18]. Prior to making dilutions, the spray-dried powder was dissolved in 0.85% (*w*/*v*) NaCl in a ratio of 1:9. The plates were then incubated at 37 °C for 48 h. After incubation, the bacteria were enumerated by expressing colony-forming units per milliliter (CFU/mL). CFU counts before and after the process were enumerated to obtain the bacterial viability rate.

Percent viability [%] = N_f_/N_r_ × 100

where N_r_ = CFU/mL before spray-drying, N_f_ = CFU/mL after spray-drying [47].

The powder was stored at 4 °C and 20 °C (±1 °C) in 50 mL sterile centrifuge tubes and then the viability of the probiotic cells was checked after 1, 2 and 4 weeks.

### 2.6. Differential Scanning Calorimetry (DSC)

The thermal properties of butters were measured using a differential scanning calorimeter DSC 2500 (TA Instruments, New Castle, DE, USA). Approximately 5–6 mg of each fatty substance was weighed in dedicated aluminum sample pans. It was heated from 10 to 100 °C (rate 10 °C/min). DSC thermal values were determined based on the average of two scans.

### 2.7. Fatty Acids Analysis

Fatty acid content was analyzed using gas chromatography, coupled with a mass spectrometer, Agilent Technologies 7890A (Agilent Technologies, Santa Clara, CA, USA), and equipped with a split/splitless-type injector. The separation was conducted with column SP^TM^ 2560, 100 m 0.25 mm ID, 0.20 µm film thickness, catalogue no. 24056 (Supelco^®^, Sigma-Aldrich Co. LLC, Saint Louis, MO, USA). Helium was used as a carrier gas at a constant flow rate of 1.2 mL/min; split 1:50. The injector temperature was set to 220 °C; detector temperature was 220 °C and furnace temperature increased from 140 °C to 240 °C at a rate of 4 °C/min. Fatty acid analysis was carried out for 45 min.

### 2.8. Moisture Content

Approximately 1 g of each powder sample was placed in an aluminum tray. The moisture determination was performed using an automatic drying oven set to 120 ± 2 °C (Radwag, Radom, Poland).

### 2.9. Water Activity

The analysis of water activity of dry microcapsules was carried out using the MS1 Set-aw, (Novasina AG, Lachen, Switzerland). Immediately after spray-drying, the 1 g of powder sample was placed in a plastic container. Water activity was measured after the samples were stabilized for 15 min at 25 °C.

### 2.10. Particle Size of Powders

The particle size of powder distribution was measured by particle size analyzer MasterSizer 2000 (Malvern Instruments, Malvern, UK) with a Scirocco 2000 dry sampling system (Malvern Instrument Ltd., Worcestershire, UK). The following parameters were used: refractive index: 1.52, vibration feed rate: 40–50%, measurement time: 15 s, 5 scans, dispersive air pressure: 4 bar. The size measurements were described as volume-weighted mean D4,3. The final results were calculated as an average of 3 sample measurements.

### 2.11. Powder Morphology

The morphology of the spray-dried powders was investigated using a scanning electron microscope (SEM) (Vega 3 LMU, Tescan, Brno, Czech Republic). The samples of powders were applied to special round metal tables using a carbon adhesive tape. Then, the tables were placed in an automatic sputter coater (Quorum Q150R S, London, UK) to cover them with a thin layer of technical gold. The surface morphology of the sample was imaged using a secondary electron detector (detector SE). The samples were imaged in the voltage range of 10 kV, and the magnification that was used was set to 2500×.

### 2.12. Color Measurement

For the color measurement, spectrophotometer CM-5 (Konica Minolta, Osaka, Japan) was used. One gram of powder was placed in a glass container, and the CIE Lab color scale was applied to measure color variation ranging from 0 to 100 for the following parameters representing typical set of colors: L*—black to white, a*—red (+) to green (−) and b*—yellow (+) to blue (−) [48].

### 2.13. Statistical Analysis

All data are presented as mean ± standard deviation (SD). Statistical significances of compared results were tested by analysis the variance (one-way ANOVA) followed by Tukey’s test. In addition to the statistical analysis in in the figure in Section 3.3 describing the effect of the type of butter used on the viability of probiotic bacteria during the drying process and storage, which was performed using Tukey’s ANOVA multiple comparison test. All statistical analysis were performed using software Statistica version 10 StatSoft Poland (Krakow, Poland). Values were considered significantly different when *p* < 0.05.

## 3. Results and Discussion

### 3.1. Differential Scanning Calorimetry

Differential scanning calorimetry (DSC) is a thermoanalytical technique that is used for studying various heat-related phenomena in complex material systems by comparing the changes in enthalpy during heating or cooling processes [49]. These phenomena in fats can be used to elucidate their physical and chemical properties with respect to thermal processes [50].

The melting enthalpy (ΔH) is related to the energy needed to melt the fat crystals contained in the samples. The melting point of fatty substances depends mainly on its fatty acid composition, and it is a significant factor affecting the embedding effect. The high content of saturated fatty acids usually appears for typical solid fat with higher melting temperature. In 2015, researchers hypothesized that during fat melting some of the thermal energy is absorbed, decreasing the internal temperature of the particles, resulting in reduced thermal shock during the spray-drying process. They have studied the melting enthalpy of pure LMF and mixed LMF: NaCas at different ratios. For pure LMF, the melting enthalpy was 87.7 [J/g] and for LMF: NaCas it increased with LMF concentration. They showed that the total melting enthalpy of the LMF/NaCas emulsion (at various *w*:*w* ratios) was positively correlated with the viability of the *Lactobacillus* LB1 isolate after spray-drying [21].

Table 2 displays the melting enthalpy values of the butters used.

**Figure 2:**
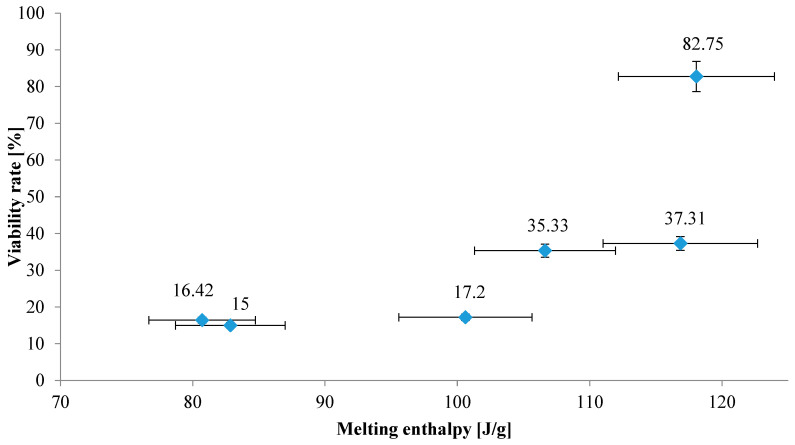
The influence of melting enthalpy of various butters on viability rate of LGG after spray-drying.

In this study, the value of the total melting enthalpy of six butter samples and their effects on the viability of bacterial cells during spray-drying were compared. For butters that did not significantly affect the percentage of live bacteria (compared to the control sample) at a level lower than 20%, no direct correlation in function of enthalpy value was observed. The melting enthalpy value of these butters was equal to or below 100 [J/g]. Whereas, for butters that significantly improved viability of bacteria, the positive correlation was observed for ΔH higher than 100 [J/g] (Figure 2). From the above, it can be concluded that above 100 [J/g] there is enough energy that can be absorbed, which makes bacterial cells less vulnerable to the adverse effects of high temperature during drying.

At the beginning of this section, it was mentioned that the higher the saturated fatty acid content, the higher the melting point usually. Table 3 presents the fatty acid profile of the butters tested. Taking into account the butters where the addition of which obtained the best results of bacterial viability, the butter with reduced fat content has a lower amount of saturated fatty acids (39.64, with H = 82.75), while salted and lactose-free butter have 50.52 and 51.95, with ΔH = 35.33 and ΔH = 37.31, respectively (Table 3).

### 3.2. The Analysis of the Fatty Acid Composition

Butter’s fatty acid composition is very important. The proportion of low- and high-melting temperature triglycerides largely determines the texture of butter. A significant amount of short-chain fatty acids with a higher content of unsaturated fatty acids makes the butter softer with a lower melting point [51].

The fatty acid composition of butters and percentage concentration of individual fatty acids in butter are presented in Table 3. The analyzed butter samples that contain the highest percentages of both C16: 0 (palmitic) and C18: 1 (oleic) acids range from 21.67% to 34.19% and from 14.78% to 27.19%, respectively. They also contain a relatively large amount of saturated C14: 0 (myristic) and C18: 0 (stearic) acids. The presence of polyunsaturated C18: 3 (linolenic) acid was determined only in salted butter (1.06%).

The largest total amount of short-chain fatty acids was found in lactose-free butter (4.31%). Reduced-fat butter contains the least monounsaturated and polyunsaturated fatty acids (16.69% and 1.38%, respectively), while clarified butter contains the most monounsaturated (30.33%) and salted butter the most polyunsaturated fatty acids (2.39%) (Table 3).

Paszczyk B. (2016) tested 10 samples of the 82% fat butter. The average percentages obtained were as follows: short chain 9.51% ± 0.81, saturated 60.65% ± 1.59, monounsaturated 26.50% ± 1.23 and polyunsaturated 3.34% ± 0.12 [52].

Taking into account the fact that the highest number of viable LGG after spray-drying was observed for systems with reduced-fat butter, as well as salted and lactose-free butter, it can be concluded that this was influenced (among other factors) by the lower content of palmitic acid (C16: 0) (compared to other butters). Butter with 60% fat content, with the addition of which the highest percentage of viability was obtained, contained the least monounsaturated and polyunsaturated fatty acids.

In another study, in which the effects of other solid fatty substances on the viability of LGG during spray-drying and storage was investigated, the fatty acid profile analysis showed that the two fats where their participation had the best survival were characterized by a significantly higher content of linoleic acid C 18 of 2–9.2% and 8.84% (taking into account the four fats where with their addition the number of viable bacteria was the highest after drying). In the case of the sample with the addition of partially hydrogenated palm oil containing a very small amount of linoleic acid C 18: 2 (0.7%), a relatively low viability rate of LGG bacteria was obtained after drying [53]. In butters, the content of linoleic acid did not differ that much and ranged from 1.34% to 1.7% (Table 3). In a 2023 study, palm stearin, which turned out to be the least effective thermoprotectant (compared to the other three fats), contains the most C 16: 0 palmitic acid and total C: 0 acids, and the least total C: 1 acids [53].

As the above analysis shows, the type and content of fatty acids in the fats used have an ambiguous effect on the survivability of LGG during the spray-drying process.

### 3.3. Viability of LGG During Spray-Drying and During Storage

**Figure 3:**
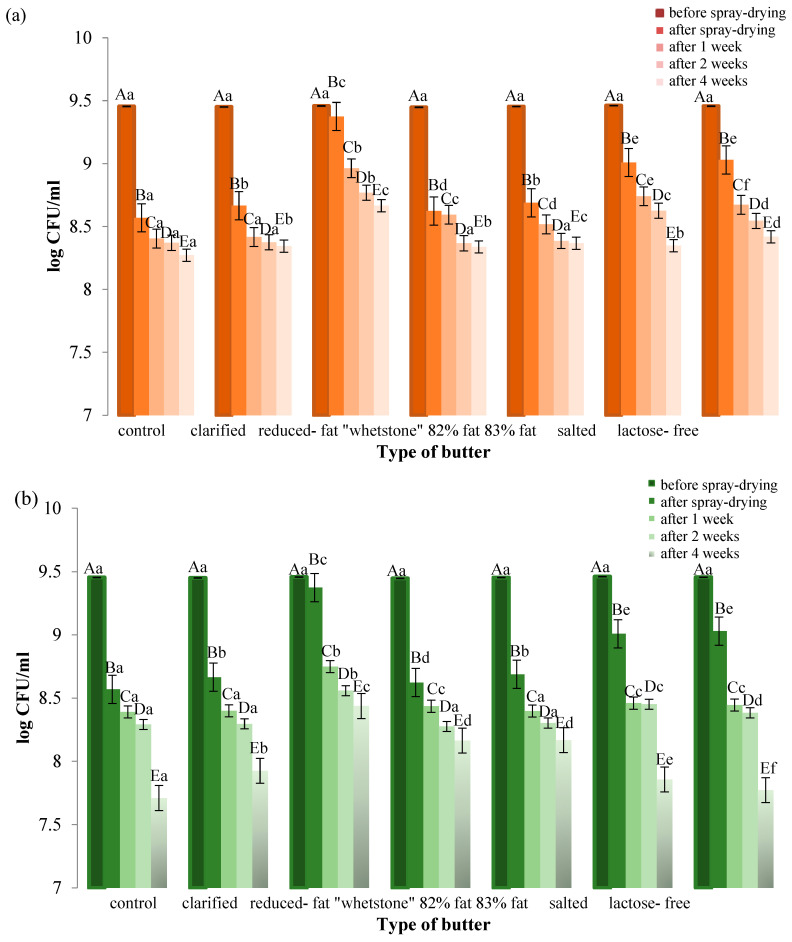
The effect of the type of butter used on the viability of probiotic bacteria during the drying process and storage: (**a**) stored at 4 °C; (**b**) stored at 20 °C (±1 °C). Values with different uppercase letters in the same column are significantly different at *p* < 0.05. Values with different lowercase letters in the same column are significantly different at *p* < 0.05.

In this study, specially prepared dispersion of LGG in emulsion of water starch Capsul^®^ HS, whey protein concentrate (WPC 80%), soy lecithin, vitamin C and various butters were spray-dried to obtain microcapsules in a form of dry powders. The number of probiotic bacteria after spray-drying and storage at 4 °C and 20 ± 1 °C for 4 weeks are presented in Figure 3a,b.

Compared to the control sample, the addition of all tested butters has a protective effect on LGG during the SD and during storage both at the refrigeration and room temperatures (20 ± 1 °C) (Figure 3a,b).

The initial bacterial concentration in all experiments was 9.45–9.46 log CFU/mL. The total number of live bacteria of the reference sample without butter after spray-drying was only 8.56 log CFU/mL. The highest number of live bacteria after drying was recorded in the case of the addition of butter with a reduced fat content (9.37 log CFU/mL), and the lowest with the addition of “whetstone” extra 82% fat content (8.62 log CFU/mL). Using clarified butter, extra butter with a fat content of 83% and a “whetstone” with a fat content of 82%, viability after drying was at a similar level to the control sample. A significantly higher number of viable cells, with respect to the sample without butter, was observed for a salted butter and lactose-free butter, with about 9.01 log CFU/mL and 9.03 log CFU/mL, respectively (Figure 3a,b).

Mizielińska et al. studied the effect of 13 emulsions with the addition of hydrophobic substances on the viability of LGG during drying and storage of powders for 4 weeks at 25 °C. The best results in storage tests were obtained for emulsions containing 20% starch, 10% cocoa butter and 10% maltodextrin and for an emulsion consisting of 20% starch with 10% cocoa butter [54].

After 4 weeks of storage of the control sample at 4 °C, the number of live cells decreased from 8.56 log CFU/mL to 8.27 log CFU/mL, and after storage at 20 ± 1 °C to 7.71 log CFU/mL. By storing the powders at a refrigerated temperature, viability in all samples was higher compared to samples stored at a room temperature (20 ± 1 °C). The highest number of live bacteria stored for 4 weeks at 4 °C was recorded using reduced-fat butter (8.67 log CFU/mL) as a protective material, and the lowest using salted butter (8.35 log CFU/mL). However, in the case of storage at 20 ± 1 °C, the best results were also obtained for butter with reduced fat content (8.44 log CFU/mL), and the lowest for lactose-free butter (7.77 log CFU/mL) (Figure 3a,b).

In 2017, the scientists who spray-dried LGG also obtained better bacterial viability by storing the powder at a lower temperature [48].

It is believed that a higher number of viable bacteria after the drying process can be obtained by lowering the outlet temperature. However, a low outlet temperature may result in a higher moisture content of the resulting powder, which may result in a greater loss of viable probiotic bacteria during storage [25].

**Figure 4:**
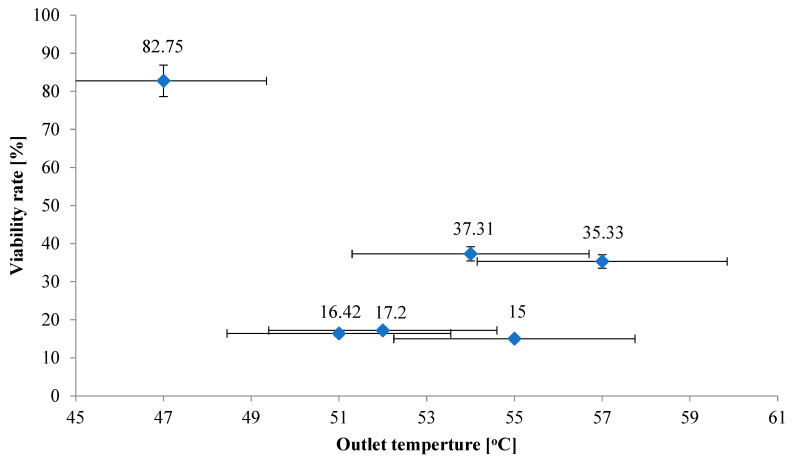
The influence of outlet temperature during spray-drying on viability of *Lacticaseibacillus rhamnosus* GG after the SD process.

During the drying process of bacteria dispersions with the addition of various butters as protective substances, different outlet air temperatures were recorded. Figure 4 shows the effect of outlet air temperature on LGG viability. During spray-drying of LGG with the addition of reduced-fat butter, which yielded the highest percentage of live bacteria (82.75%), the outlet air temperature was 47 °C. During drying of samples with the addition of butters that did not significantly improve cell viability (15–17.2%), the outlet temperature was higher and ranged from 51 °C to 55 °C; however, in the case of using the other two butters, the temperatures were 54 °C and 57 °C, and the viability rates were 37.31% and 35.33%, respectively. Therefore, no direct correlation was observed; however there are some general trends where lower outlet temperature, especially below 50 °C, results in the highest viability of LGG. Apparently, the overall relationship to process conditions is more complex and also depends on some other factors.

As shown in Table 4, the selected butters differed not only in the fat content, but also in the content of fatty acids, carbohydrates (including sugars), proteins and salt.

Viability of bacteria in the powder containing clarified butter with higher content of fats and saturated fatty acids, and the lower of carbohydrates (including sugars), protein and salt, was at a similar level as in the case of “whetstone” and extra butter. On the other hand, reduced-fat butter, containing a lower content of fat and saturated fatty acids, and higher content of other ingredients (except salt) (Table 4), indicates the highest percentage of living LGG after spray-drying.

Despite how the following “whetstone”, extra, salted and lactose-free butters have a similar content of all ingredients (except salt—salted butter has as much as 1.8 wt.%, and the rest have 0.2 wt.% or lower), there is no impact of these components on bacterial viability during the spray-drying process (Table 4).

**Figure 5:**
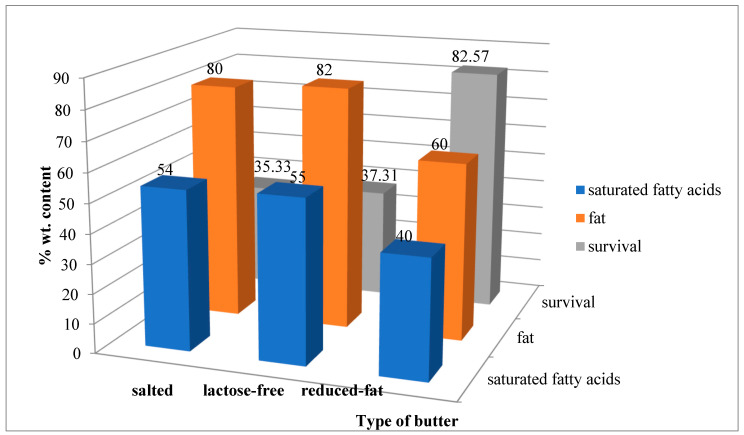
Content (% wt.) of fat, saturated fatty acids and bacterial viability after spray-drying for three butter samples.

A significantly higher percentage of viable cells after spray-drying with respect to the control sample was observed for salted butter, lactose-free butter and reduced-fat butter, at approx. 35.4%, 37.3% and 82.6%, respectively. Salted and lactose-free butters contain a higher content of fat and saturated fatty acids. However, for butter with reduced fat content, the highest viability rate was obtained (Figure 5). At the same time, based on the product specification, this butter has the highest content of carbohydrates, including sugars and proteins, compared to salted and lactose-free butter.

It is assumed that the highest viability of LGG after the spray-drying process may be indirectly influenced by the specific correlation between the higher content of proteins and sugars and the lowest content of fats and fatty acids in reduced-fat butter, compared to other butters.

### 3.4. The Moisture Content and Water Activity

The values of moisture content and water activity in dry powder microcapsules formulated with adding different butters are displayed in Table 5.

In this study, the moisture content of all samples was found to be within the acceptable limits for food powders (<5%) resulting good shelf-life stability [11].

The water content of the reference sample was 4.04 ± 0.035. In powders with butters, it was from 3.51 ± 0.01 (“whetstone” extra 82% fat content) to 4.57 ± 0.04 (clarified butter). The moisture content values of samples with addition of reduced-fat butter, “whetstone” extra 82% fat content and extra 83% fat content do not differ significantly from the reference sample (*p* > 0.05), while powders with addition of clarified, salted and lactose-free butter differ significantly from reference sample (*p* < 0.05). All samples with the addition of butter had a lower water content than the reference sample, except for the sample with the addition of clarified butter. The water content values of powders with the addition of extra 83% fat content butter and salted butter, and reduced-fat and lactose-free butter are also similar, at 3.73 ± 0.031 and 3.77 ± 0.03, and 3.88 ± 0.026 and 3.90 ± 0.01, respectively. Taking into account the butters where their addition showed no significant improvement in bacterial survival after drying (compared to the control sample), and thus these values were similar, the moisture content of the obtained powders differed from each other. Namely, the moisture contents in the powder with the addition of clarified butter, “whetstone” extra 82% fat content and extra 83% fat content were 4.57 ± 0.04, 3.51 ± 0.01 and 3.73 ± 0.031, respectively.

A value of water activity is an index of great importance due to its effect on the shelf-life of powder. A high water activity is associated with faster biochemical reactions, and consequently, a shorter shelf-life [42].

The a_w_ of the reference sample was 0.120 ± 0.002, whereas microcapsules containing different types of butter were characterized by slightly higher values of water activity from 0.125 ± 0.004 (with reduced-fat butter) to 0.174 ± 0.002 (with clarified butter). Powders with the addition of reduced-fat butter with 60% total fat content (the addition of which had the best result of LGG viability after spray-drying), “whetstone” extra of 82% (addition of which obtained the lowest number of live bacteria after drying) and butter extra of 83% had a very similar values of water activity 0.125 ± 0.004, 0.127 ± 0.006 and 0.128 ± 0.003, respectively. The a_w_ value of powders with the addition of all butters differ significantly from the reference sample (*p* > 0.05).

No direct correlation was observed between water activity or powder moisture and bacterial viability.

Similar results were obtained by other scientists in 2015 for the three spray-dried *Lactobacillus* isolates in 10% *w*/*v* sodium caseinate (NaCas) in the presence of either LMF or vegetable oil with the ratio of oil or LMF to NaCas at 1:1. In both types of formulated microcapsules, the water contents in powders were approx. 3.3 and 3.7 wt.%, and the water activities were 0.19 and 0.20 for oil+ NaCas and LMF + NaCas, respectively [21].

A moisture content below 4% was also obtained for all samples containing *Bifidobacterium* BB-12 described by researchers in 2018. They microencapsulated *Bifidobacterium* BB-12 using full-fat goat’s milk and/or inulin and/or oligofructose as the carrier material. For powder with the addition of full-fat goat’s milk, full-fat goat’s milk powder and inulin, full-fat goat’s milk and oligofructose and full-fat goat’s milk/inulin/oligofructose, they obtained moisture contents of 3.68 ± 0.15, 3.48 ± 0.13, 3.46 ± 0.15 and 3.42 ± 0.19, respectively. Also, here the water activity values were very similar, at levels of 0.208 ± 0.004, 0.148 ± 0.007, 0.179 ± 0.002 and 0.179 ± 0.001, respectively [55]. Soukoulis et al., tested three different milk proteins (skimmed milk powder, sodium caseinate and whey protein concentrate) for their ability to stabilize microencapsulated L. acidophilus by spray-drying. They tested combinations of all carrier materials with added glucose and trehalose. The water content obtained ranged from 1.66 ± 0.04 to 4.19 ± 0.03 and a_w_ value from 0.10 ± 0.00 to 0.27 ± 0.02 [56].

### 3.5. Particle Size and Powders Morphology

**Figure 6:**
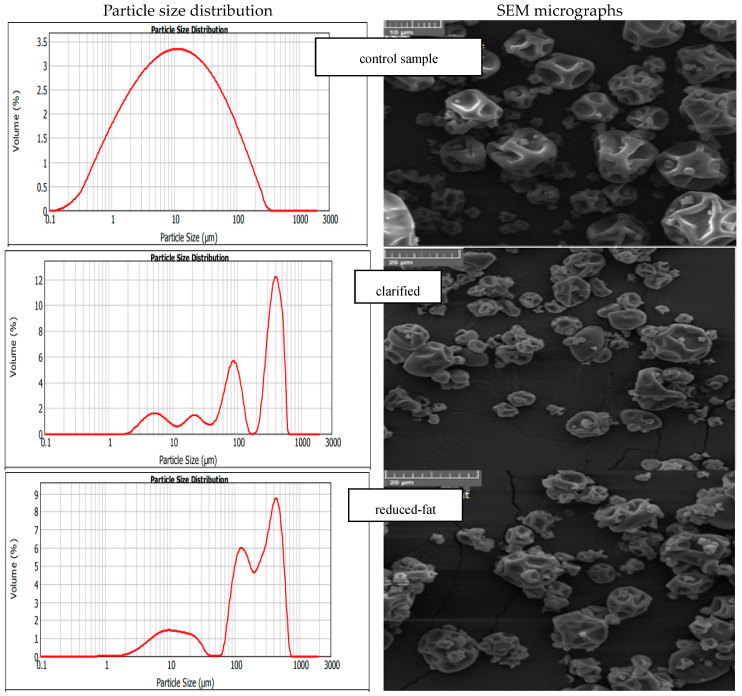

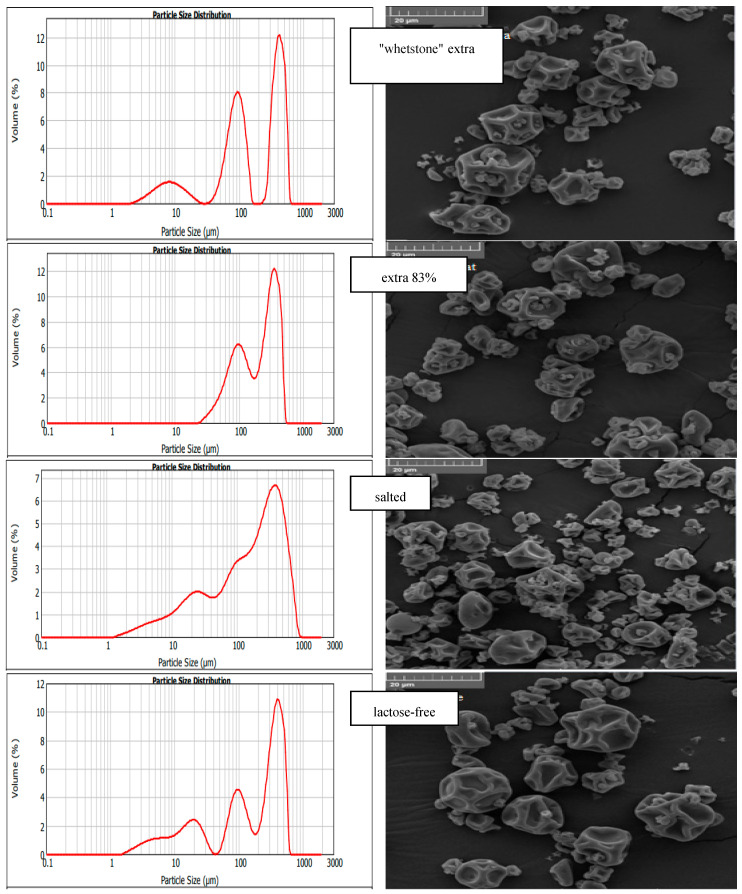
Particle size distribution and SEM micrographs of powders with different butters.

The particle mean size distribution D_4,3_ values of the microcapsules are shown in Table 6. The average particle size of the reference sample was 29.43 ± 2.06 µm. Surprisingly, all particles with the addition of butters were characterized by much larger sizes; however, their sizes were close to each other and within the range from 220.85 ± 7.04 to 232.78 ± 1.37. The highest value was obtained for the powder with the addition of clarified butter, and the lowest was noticed for the powder containing reduced-fat butter. In Figure 6, it is observed that the control sample consists of particles of similar size, while in the powders with the addition of butters, at least two fractions are visible—smaller, single particles, and larger ones that form agglomerates. The addition of butters caused the powders to show an increased cohesion between the particles, which means that the system is unable to disperse them properly or they have the tendency to stick together during drying.

The effect of the ingredients can modify the particles mean size, e.g., high molecular weight materials can increase the spray-dried particle size [57]. However, we believe that in this case there was another phenomenon during this measurement that caused such a huge difference in the obtained mean diameter values between fat-containing and pure reference samples. In this study, the large particle size of powders containing various butters may be related to aggregate formation.

Materials that are insoluble in water tend to form agglomerates when dried [58]. As shown in Figure 6, the differences in the particle size of powders containing butters (in individual samples) are significant, therefore, here, we used the microscopic observations for final confirmation of the real size of obtained particles.

The average particle size of the microcapsule can have an influence on its stability and efficiency of microencapsulation. Larger powders generally result in more protection of the sensitive material, but they have poor dispersibility in products. However, particles that are too small may be hollow and, therefore, in this case the final microencapsulation efficiency may be very low [59].

We did not observe a direct correlation between powder particle size and LGG cell viability during either the drying process or storage at either temperature.

Figure 6 shows the scanning electron microscopy (SEM) micrographs of the spray-dried powders containing LGG with addition of different butters. The microcapsules containing different butters were similar in appearance, indicating that the butters used did not affect the morphology and size of the individual particles. They are also very similar to the control sample.

No cracks were noticed on the surface, which proves the low permeability of gases and the lack of possibility for bacteria to “get out” to the surface of the microcapsules and, as a result, to protect the LGG by effective full encapsulation as a result of spray-drying. Santos et al. used palm oil (melting point 30–40 °C), which melts easily and hardens rapidly at room temperature. Palm-oil-coated microcapsules were found to have virtually no pores or cracks on their surface, which may help maintain the integrity of the capsules during storage [60].

The particles were mostly spherical in shape, and the surfaces were wrinkled with concavities. The “flat ball” effect has been also reported by other researchers in 2005. It is directly associated with the conditions during spray-drying (particularly with the heat penetration and rapid evaporation of the water from the liquid droplet) [61].

The SEM images also showed a similar size of powder particles, both with the addition of butters and the control sample. Particles of different sizes are observed in the individual images, and it can be observed that the smaller particles “fill” some of the cavities created in the larger particles.

The SEM pictures of obtained particles indicate that all microcapsules in powder form are much smaller in size than those measured with the MasterSizer using the LSD technique. It is assumed that the dispersion energy of the flowing air used during the LSD measurements was too small to overcome the cohesive forces between the powder particles. Therefore, it is important to use both the MasterSizer and SEM for particle size analysis and comparison when testing powders containing lipid substances.

### 3.6. Color Measurement

The color of spray-dried powders is an important quality attribute. Color can be affected by powder structure, the color of the spray-drying carrier and drying conditions, such as high inlet temperature or drying time [47]. Table 7 displays the color parameters of the microencapsulated powders. The L* for the lightness from black (0) to white (100), positive a* value indicates red coloration, and positive b* value indicates yellow coloration of the powder. The control sample was the brightest (89.64 ± 0.01). The powder containing lactose-free butter had a very similar level of brightness (89.32). Regardless of the type of butter added, the powders were brighter. All samples were characterized by a positive a* value suggesting a tendency to red color hues and positive b* values indicating yellowish hue color. In the case of powders containing butter, an increase in the a* value is observed (even more than twice with the addition of clarified butter, or 2.5 times with lactose-free butter). However, taking into account the b* value, a lower value is observed in the powders with the addition of butter than in the control sample (Table 7).

In our previous work, we used other solid fats as protective fats, namely, palm oil; partially hydrogenated palm oil; palm stearin; chemically interesterified fully hydrogenated palm kernel oil; chemically interesterified palm oil; chemically interesterified mixture of palm kernel oil, coconut oil and palm stearin; hydrogenated soybean oil and shea butter. We obtained similar results. The L* value ranged from 85.57 (with added shea butter) to 89.32 ± 0.01 (for chemically interesterified palm oil), the a* value from 0.1 (chemically interesterified mixture) to 0.62 (with added palm oil) and the b* value ranged from 10.68 ± 0.01 (chemically interesterified fully hydrogenated palm oil) to 12.95 (sample with added shea butter) [53].

Similar results have been also reported by other scientists in 2021 with positive both a* and b* values for spray-dried powder containing *Bifidobacterium* BB-12 in matrixes based on lactose-free skim milk powder, lactose-free skim milk powder and inulin, and lactose-free skim milk powder and oligofructose [62].

## 4. Conclusions

The addition of fatty substances in the form of various types of butter has been shown to have a protective effect on LGG bacteria during the spray-drying process and storage both at 4 °C and 20 ± 1 °C (compared to the control sample). Stored powders are more stable at 4 °C (viability in all samples was higher).

Both the moisture content and the water activity value in all samples are within acceptable limits for food powders, which contributes to good shelf-life stability during storage, but no direct effect of these parameters on LGG viability rate was observed.

It is assumed that the highest viability of probiotic bacteria after the spray-drying process and after 4-week storage at 4 °C and at 20 ± 1 °C may be indirectly influenced by the relationship between the appropriate higher content of proteins and sugars and with lower content of fats and fatty acids in butter with a reduced fat content, compared to other butters used.

For the three butters that significantly improve viability the relationship, it was observed that the higher the melting enthalpy, the higher the percentage of live probiotic cells after spray-drying.

Taking into account the fact that the highest number of viable LGG after spray-drying was observed for systems with reduced-fat butter, as well as salted and lactose-free butter, it can be concluded that this was influenced (among other factors) by the lower content of palmitic acid (C16: 0) (compared to other butters). Butter with 60% fat content obtained the highest percentage of viability when added and contained the least monounsaturated and polyunsaturated fatty acids.

The results presented in the publication confirmed the protective role of selected fatty food products on the survival of probiotic bacteria LGG both during both the spray-drying process and their storage in typical food storage conditions, which indicates the possibility of using them in industrial processes to increase the durability of additives and products using probiotic powders obtained by spray-drying.

The protective additives proposed in this work have great application potential, as the results indicate that the use of even a relatively small addition of various types of butter improves the survival of bacteria and the properties of the obtained powders, which may be important for the creation of products with a longer survival period.

In addition, the cost of selected natural butters is relatively low, and the process is practically no different from the classic spray-drying process and the production of powders with the participation of probiotic bacteria.

Additional studies using model fatty substances with various melting temperatures still need to be performed in order to identify and verify these complex relationships in the case of application of natural fatty products such as butters as protectants during spray-drying process of living bacteria.

## Figures and Tables

**Table 1:** Emulsion composition.

Component	[%] (*w*/*v*)
Starch Capsul^®^ HS	15
80% WPC	5
Lecithin	0.6
Ascorbic acid	0.6
Butter	6

**Table 2:** Melting enthalpy values of butters.

Type of Butter	[J/g]
clarified	80.71 ± 0.01 ^a^
reduced-fat	118.07 ± 0.10 ^b^
“whetstone”extra 82% fat content	82.84 ± 0.06 ^c^
extra 83% fat content	100.60 ± 0.002 ^d^
salted	106.66 ± 0.04 ^e^
lactose-free	116.86 ± 0.08 ^f^

Values are means ± standard deviation of duplicate determinations. Values with different lowercase letters in the same column are significantly different at *p* < 0.05.

**Table 3:** The fatty acid composition and their percentage concentration in butter samples.

Type of Fatty Acid	Type of Butter and Fatty Acid Concentrations [%]					
	Clarified	Reduced-Fat	“Whetstone”Extra 82% Fat Content	Extra 83% Fat Content	Salted	Lactose-Free
4:0	0.477	0.247	0.443	0.464	0.386	0.598
6:0	0.710	0.359	0.514	0.565	0.516	0.745
8:0	0.673	0.433	0.590	0.537	0.522	0.707
10:0	2.157	1.256	1.743	1.760	1.729	2.255
Ʃ short-chain	4.016	2.295	3.290	3.327	3.152	4.305
12:0	3.037	3.122	4.116	2.648	2.687	3.129
14:0	11.933	7.376	10.095	10.575	10.291	10.620
15:0	1.201	0.667	0.848	1.061	0.995	1.043
16:0	34.185	21.668	30.356	30.766	25.933	28.415
18:0	13.395	6.804	9.310	10.042	10.614	8.746
Ʃ saturated	63.751	39.638	54.726	55.091	50.520	51.954
14:1	1.235	0.727	1.024	1.194	1.231	1.167
16:1	1.928	1.187	1.593	1.805	1.658	1.838
18:1	27.168	14.777	19.819	20.170	21.046	21.046
Ʃ monounsaturated	30.330	16.691	22.437	23.169	23.935	24.051
18:2	1.703	1.376	1.547	1.412	1.335	1.690
18:3	-	-	-	-	1.057	-
Ʃ polyunsaturated	1.703	1.376	1.547	1.412	2.392	1.690

**Table 4:** Butter composition and viability of LGG after their addition to spray-dried emulsions.

Type of Butter	Fat [%]	Saturated Fatty Acids [g/100 g]	Carbohydrates (Including Sugars) [g/100 g]	Protein [g/100 g]	Salt [g/100 g]	Other Ingredients	Viability Rate [%]
clarified	99.8	65	0.1 (0.1)	0.1	0	lack of information	16.42
reduced-fat	60	40	2.0 (2.0)	1.5	0.2	pasteurized cream	82.57
“whetstone”extra 82% fat content	82	57	1.0 (0.7)	0.7	<0.02	pasteurized cream	15
extra 83% fat content	83	54	0.8 (0.8)	0.6	0	cream	17.2
salted	80	54	0.6	0.6	1.8	cream, salt	35.33
lactose-free	82	55	0.7 (0.7)	0.7	0	lack of information	37.31

**Table 5:** Water content and water activity of spray-dried powder.

Type of Butter	Water Content	a_w_
reference sample	4.04 ± 0.035 ^a^	0.120 ± 0.002 ^a^
clarified	4.57 ± 0.04 ^b^	0.174 ± 0.002 ^b^
reduced-fat	3.88 ± 0.026 ^a^	0.125 ± 0.004 ^c^
“whetstone” extra 82% fat content	3.51 ± 0.01 ^a^	0.127 ± 0.006 ^d^
extra 83% fat content	3.73 ± 0.031 ^a^	0.128 ± 0.003 ^e^
salted	3.77 ± 0.03 ^c^	0.138 ± 0.004 ^e^
lactose-free	3.90 ± 0.01 ^c^	0.141 ± 0.007 ^c^

Values are means ± standard deviation of triplicate determinations. Values with different lowercase letters in the same column are significantly different at *p* < 0.05.

**Table 6:** The particle size distribution of powders.

Type of Butter	D_4,3_ [µm] of Powders
reference sample	29.43 ± 2.06 ^a^
clarified	232.78 ± 1.37 ^b^
reduced-fat	220.85 ± 7.04 ^c^
“whetstone” extra 82% fat content	226.14 ± 4.46 ^d^
extra 83% fat content	232.43 ± 3.63 ^b^
salted	229.53 ± 1.37 ^e^
lactose-free	225.19 ± 2.29 ^f^

Values are means ± standard deviation of triplicate determinations. Values with different lowercase letters in the same column are significantly different at *p* < 0.05.

**Table 7:** The effect of the type of butter on powder color.

Type of Butter	L*	a*	b*
control sample	89.64 ± 0.01 ^a^	0.26 ± 0.01 ^a^	13.7 ± 0.01 ^a^
clarified	88.61 ± 0.01 ^b^	0.58 ± 0.01 ^b^	12.51 ± 0.01 ^b^
reduced-fat	88.87 ± 0.01 ^c^	0.39 ± 0.01 ^c^	11.31 ± 0.01 ^c^
“whetstone” extra 82% fat content	87.89 ± 0.00 ^d^	0.45 ± 0.01 ^d^	11.21 ± 0.01 ^d^
extra 83% fat content	87.46 ± 0.00 ^e^	0.41 ± 0.01 ^c^	12.58 ± 0.01 ^e^
salted	88.55 ± 0.00 ^f^	0.31 ± 0.00 ^e^	11.36 ± 0.00 ^f^
lactose-free	89.32 ± 0.00 ^g^	0.65 ± 0.01 ^f^	12.74 ± 0.00 ^g^

The average values of 5 measurements with standard deviation. L* for the lightness from black (0) to white (100). a* from green (−) to red (+). b* from blue (−) to yellow (+). Values with different lowercase letters in the same column are significantly different at *p* < 0.05.

## Data Availability

The original contributions presented in the study are included in the article, further inquiries can be directed to the corresponding author.
